# Maternal pre-pregnancy obesity modifies the association between first-trimester thyroid hormone sensitivity and gestational Diabetes Mellitus: a retrospective study from Northern China

**DOI:** 10.1186/s13098-023-01188-6

**Published:** 2023-10-25

**Authors:** Honglin Sun, Yibo Zhou, Jia Liu, Ying Wang, Guang Wang

**Affiliations:** 1 Department of Endocrinology, Beijing Chao-yang Hospital, Capital Medical University, Beijing, 100020 China; 2 Physical Examination Center, Beijing Chao-Yang Hospital, Capital Medical University, Beijing, 10020 China

**Keywords:** Sensitivity to thyroid hormones, Pre-pregnancy obesity, TFQI, GDM

## Abstract

**Background:**

Contradictory relationships have been observed between thyroid function and gestational diabetes mellitus (GDM). Previous studies have indicated that pre-pregnancy BMI (pBMI) could modify their relationships. Few studies have illustrated the role of thyroid hormone sensitivity on GDM. We aimed to explore the effect of pre-pregnancy obesity on the association between early pregnancy thyroid hormone sensitivity and GDM in euthyroid pregnant women.

**Methods:**

This study included 1310 women with singleton gestation. Subjects were classified into pre-pregnancy obese and non-obese subgroups by pBMI levels with a cutoff of 25 kg/m^2^. Sensitivity to thyroid hormone was evaluated by Thyroid Feedback Quartile-Based Index (TFQI), Chinese-referenced parametric TFQI (PTFQI), TSH Index (TSHI) and Thyrotrophic T4 Resistance Index (TT4RI). The associations between these composite indices and GDM were analyzed using multivariate regression models in the two subgroups, respectively.

**Results:**

In pre-pregnancy non-obese group, early pregnancy TFQI, PTFQI, TSHI and TT4RI levels were higher in subjects with incident GDM compared to those without GDM (all *P* < 0.05). By contrast, obese women with GDM exhibited lower levels of those indices (all *P* < 0.05). The occurrence of GDM were increased with rising TFQI, PTFQI, TSHI and TT4RI quartiles in non-obese women ( all *P* for trend < 0.05), while exhibited decreased trend across quartiles of those indices in obese women (all *P* for trend < 0.05). Further logistic analysis indicated contrary relationships between thyroid hormone sensitivity and the occurrence of GDM in the two groups, respectively. The OR of the fourth versus the first quartile of TFQI for GDM was 1.981 (95% CI 1.224, 3.207) in pre-pregnancy non-obese group, while was 0.131 (95% CI 0.036, 0.472) in pre-pregnancy obese group. PTFQI and TSHI yielded similar results.

**Conclusions:**

The association between maternal sensitivity to thyroid hormones during early gestation and the occurrence of GDM was modified by pre-pregnancy obesity.

**Supplementary Information:**

The online version contains supplementary material available at 10.1186/s13098-023-01188-6.

## Background

Maternal thyroid function during the first trimester plays a vital role in maternal metabolism [1–6]. Numerous studies have disclosed definite influences of abnormal first-trimester thyroid function on adverse maternal complications [1, 3, 4, 7–13]. Moreover, recent studies indicated that even variation in thyroid parameters within the normal range during the first trimester was related to adverse pregnancy outcomes [14–16].

Recently, The indices for assessment of central sensitivity to thyroid hormones, namely Thyroid Feedback Quantile-Based Index (TFQI), parametric TFQI (PTFQI), TSH index (TSHI) and thyrotrophic T4 resistance index (TT4RI), which were calculated by combination of both TSH and FT4, have proven to be more representative of thyroid homeostasis than single parameter [17–19]. Growing studies have revealed the association between these composite indices and metabolic-related diseases such as diabetes, hypertension and NAFLD, even in euthyroid populations [17, 19–21]. Our previous studies also revealed close links between those indices and lipid disorders in euthyroid populations [22, 23]. However, limited studies have evaluated those composite indices with pregnancy outcomes, especially those related to maternal energy balance, such as gestational diabetes mellitus (GDM).

As a common obstetric metabolic disorder, GDM poses both short- and long-term threats to maternal and child health [11, 24]. So far, contradictory conclusions have been drawn to illustrate the causality between thyroid function and the occurrence of GDM [11, 25, 26]. For example, both hypothyroidism (overt/subclinical/isolated) and hyperthyroidism have been reported to be associated with GDM [9, 10, 27]. Some studies have indicated adverse relationships of FT4, TSH or both with GDM under euthyroid status [4, 11, 25], whereas other studies indicated contrary relationships [7, 11, 25]. These inconsistencies indicated complex relationships between the thyroid system and glucose homeostasis, which could hardly be explained by a single parameter. Although central thyroid hormone sensitivity has been illustrated to be closely associated with DM in non-pregnant population, few studies have explored their relationships in pregnant women.

The pre-pregnancy body mass index (pBMI) is a well-known determinant on pregnancy outcomes [28, 29]. Pregnancy obesity is an important risk factor for GDM [5, 30, 31]. Furthermore, previous studies have indicated a modifying role of pBMI on the association between thyroid hormones and pregnancy outcomes [29, 32]. It remains unclear whether pBMI could also modify the effect of sensitivity to thyroid hormones during early pregnancy on GDM. Therefore, our aim was to assess the relationships between sensitivity to thyroid hormones and GDM under pre-pregnancy obese and non-obese status, and further decipher the effect of pre-pregnancy obesity on their relationships in euthyroid women from Northern China.

## Materials and methods

### Study population

**Fig. 1:**
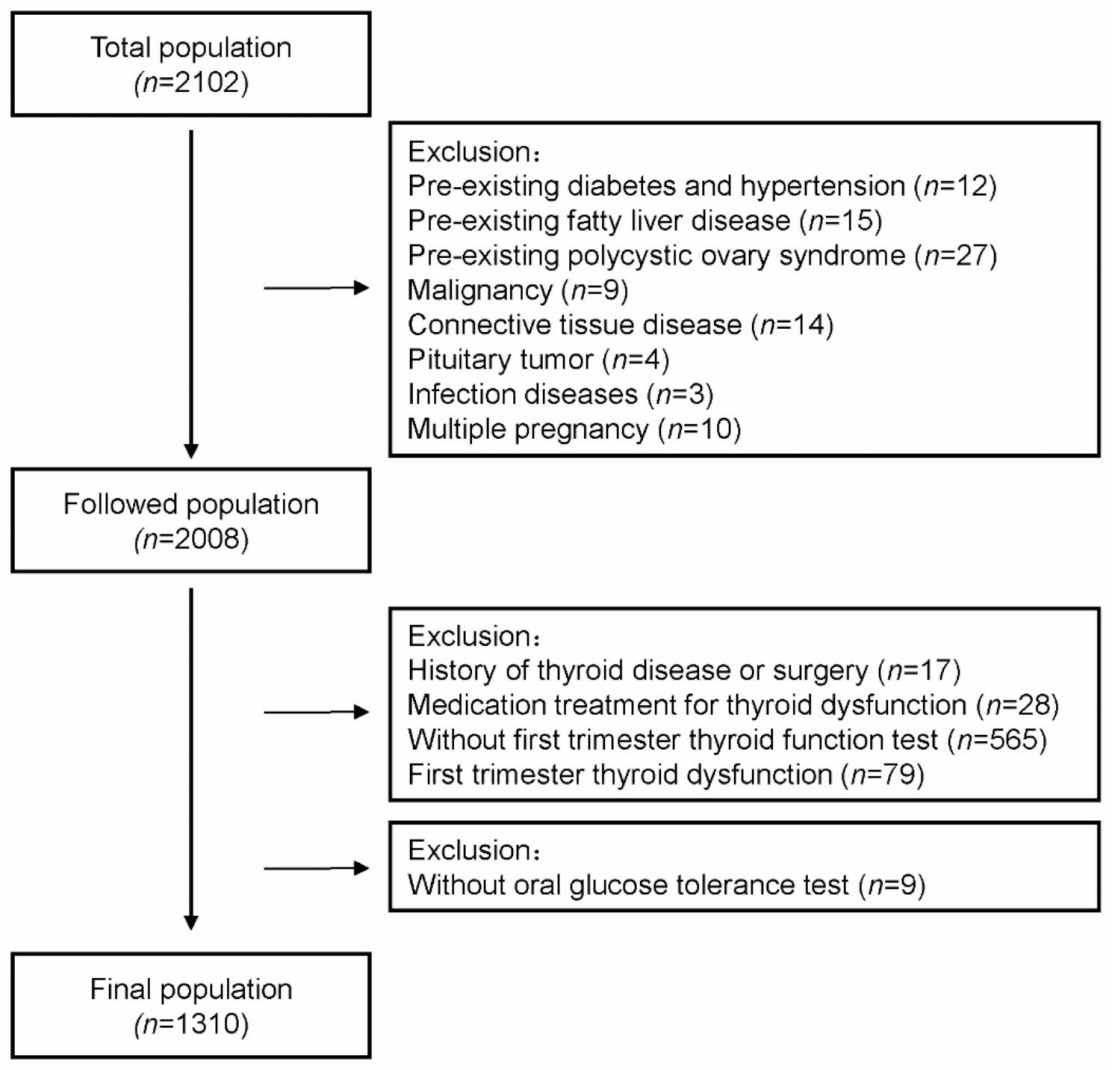
The flow chart of the study population

2102 pregnant women who underwent prenatal check-ups and delivery during January 2020 and December 2021 at Beijing Chao-yang Hospital were enrolled in this study. Subjects without OGTT results (*n* = 9), with pre-existing diabetes and hypertension (*n* = 12), with malignancy (*n* = 9), pituitary tumor (*n* = 4), infection diseases (*n* = 3), polycystic ovary syndrome (PCOS) (*n* = 27), fatty liver disease (FLD) (*n* = 15), connective tissue disease (CTD) (*n* = 14), multiple pregnancies (*n* = 10), without thyroid function test during first trimester (*n* = 565), with thyroid disease/surgery histories (*n* = 17), with thyroid (interfering) medication usage (*n* = 28), and with abnormal thyroid function (*n* = 79 ) were excluded. The final analysis contained 1310 individuals (Fig. 1). Ethical approval was obtained from the Ethics Committee of Beijing Chao-yang Hospital (Approval number: 2022 − 517). All participants signed the written informed consent.

### Data Collection and parameters measurements

Medical history, drug usage, maternal age, parity, pre-pregnancy weight and height were obtained during first clinical visit. Blood samples were collected during the first trimester (between 9 and 13 weeks) after overnight fasting. Maternal serum FT3, FT4, TSH, anti-thyroglobulin antibodies (TG-Ab) and anti-thyroid peroxidase antibody (TPO-Ab) were measured with chemiluminescence immunoassay (ADVIA Centaur XP, Siemens Healthcare Diagnostics, Germany). Biochemical parameters including triglyceride (TG), total cholesterol (TC), high-density lipoprotein cholesterol (HDL-C), low-density lipoprotein cholesterol (LDL-C), alanine aminotransferase (ALT), creatinine, aspartate aminotransferase (AST) and uric acid (UA) were detected with Siemens Advia 2400 autoanalyzer (Siemens Healthcare Diagnostics, Germany). The OGTT test was conducted during the third trimester (24–28 weeks) by oral intake of a 75 g glucose load after fasting overnight. Plasma glucose levels were detected before and during 1 and 2 h of the OGTT test.

### Variables definition

The guidelines on diagnosis and management of thyroid disease during pregnancy and postpartum by Chinese Medical Association recommended to establish population-based, trimester-specific, and assay method-specific thyroid hormone reference intervals for gestational women [33, 34]. Here, euthyroid during early pregnancy was defined as TSH (0.13–3.93 µIU/mL) and FT4 (12.00-23.34 pmol/L) within the reference ranges according to a previous national survey conducted in Chinese pregnant women by using the same thyroid hormones detection method [33, 35]. TFQI = cumulative distribution function (cdf) FT4 – (1 – cdf TSH). PTFQI = Φ((FT4 − µFT4)/σFT4) − (1 − Φ((ln TSH − µLn TSH)/σln TSH)), where µFT4 = 16.1372 (pmol/L), σFT4 = 1.90549, µln TSH = 0.1013 (µIU/mL), and σln TSH = 0.66922 for the Chinese pregnant women during the first trimester. TSHI, TT4RI and FT3/FT4 ratio were calculated as described previously [22].

pBMI was calculated as previously described [5]. Participants were classified into obese and non-obese subgroups according to the WHO classification for Asian population with a pBMI cutoff of 25 kg/m^2^ [36]. GDM was diagnosed if fasting plasma glucose ≥ 91.8 mg/dL or 1 h plasma glucose levels ≥ 180.0 mg/dL or 2 h glucose levels ≥ 153.0 mg/dL based on the OGTT test [24]. eGFR, pre-pregnancy hypertension, diabetes and FLD were defined according to previous studies [37].

### Statistical analysis

Continuous variables were expressed as mean ± standard deviation (SD) or median (upper and lower quartiles) following normality detection by Shapiro-Wilk test. Categorical variables were expressed as number (proportion). Data difference between two groups were compared by unpaired Student’s *t* test, Mann-Whitney U test or the Chi-square test.

The correlation between indices of thyroid system and plasma glucose concentration during OGTT test were detected by Spearman analysis. Logistic regression analyses was conducted to explore the associations between per SD increase of thyroid indices and GDM in pre-pregnancy obese and non-obese women, separately. Model 1 was without adjustment, Model 2 was adjusted for age, parity, first trimester TG, LDL-C, UA and eGFR. The interactions between thyroid sensitivity indices and pre-pregnancy obesity on the occurrence of GDM were further analyzed. The four composite Indices were then divided into quartiles (Q) (TFQI:Q1 < -0.24, -0.24 ≤ Q2 < -0.01, -0.01 ≤ Q3 < 0.24, 0.24 ≤ Q4; PTFQI: Q1 < -0.23, -0.23 ≤ Q2 < 0.00, 0.00 ≤ Q3 < 0.25, 0.25 ≤ Q4;TSHI:Q1 < 1.89, 1.89 ≤ Q2 < 2.35, 2.35 ≤ Q3 < 2.72, 2.72 ≤ Q4; TT4RI:Q1 < 12.00, 12.00 ≤ Q2 < 19.58, 19.58 ≤ Q3 < 28.01, 28.01 ≤ Q4). The linear trends of the proportions of GDM across thyroid indices quartiles were analyzed by the Cochran Armitage trend test. The associations of thyroid parameters quartiles with GDM were explored by binary logistic regression analyses adjusted for age, parity, first-trimester TG, LDL-C, UA and eGFR.

The IBM SPSS Statistics software, version 27 (IBM Corporation, Armonk, NY, USA) was applied in the current analysis and two-tailed *P* value < 0.05 were considered statistically significant for this study. Smoking and drinking were not taken as confounders due to the small proportion of women with smoking or alcohol consumption habits [29].

## Results

### Basic characteristics of the study population

As shown in Table 1, the proportion of GDM was 17.2% for the whole population. Compared to non-GDM group, the GDM group exhibited older age, higher pBMI levels, shorter gestational week and higher first-trimester TG, TC, LDL-C levels (all *P* < 0.001). There were no statistical difference in composite indices of thyroid hormone sensitivity between women in GDM and non-GDM groups. The subjects were the categorized into pre-pregnancy non-obese and obese subgroups according to pBMI levels (Supplementary Table 1). Compared to pre-pregnancy non-obese group, the obese group exhibited not only older age, longer gestational week, higher first-trimester TG, TC, LDL-C levels but also higher proportion of GDM (all *P* < 0.05). Although TSHI and TT4RI were higher in obese group compared to non-obese group, no statistical difference of the TFQI and PTFQI was observed between the two groups.

**Table 1:** Basic characteristic of the population with and without GDM.

Variables		Total		Non-GDM		GDM	*P* value
*N*		1310		1085		225	
Age (years)		31.62 ± 3.63		31.35 ± 3.48		32.89 ± 4.07	< 0.001
Gestational time (week)		39.23 ± 1.29		39.29 ± 1.30		38.96 ± 1.19	< 0.001
pBMI (kg/m2)		21.63 ± 3.00		21.46 ± 2.96		22.46 ± 3.08	< 0.001
Parity, n (%)							0.131
≥ 1		277 (21.1%)		221 (20.4%)		56 (24.9%)	
0		1033 (78.9%)		864 (79.6%)		169 (75.1%)	
Thyroid parameter							
FT3 (pmol/L)		4.93 ± 0.49		4.93 ± 0.49		4.95 ± 0.48	0.468
FT4 (pmol/L)		16.12 ± 1.90		16.12 ± 1.89		16.10 ± 1.96	0.872
TSH (µIU/mL )		1.22 (0.75,1.79)		1.21 (0.74, 1.78)		1.28 (0.84, 1.93)	0.191
FT3/FT4 ratio		0.309 ± 0.037		0.308 ± 0.036		0.311 ± 0.038	0.368
TFQI		0.00 ± 0.36		-0.01 ± 0.36		0.02 ± 0.35	0.342
PTFQI		0.01 ± 0.35		0.00 ± 0.35		0.03 ± 0.34	0.320
TSHI		2.35 (1.89, 2.72)		2.34 (1.87, 2.71)		2.37 (1.97, 2.76)	0.193
TT4RI		19.58 (12.00, 27.97)		19.47 (11.79, 27.75)		20.08 (14.05, 29.34)	0.193
TPO-Ab (U/L)^*^		30.10 (14.00, 41.70)		30.00 (14.00, 41.70)		30.95 (14.00, 41.10)	0.639
TG-Ab (U/L)^#^		16.55 (7.50, 27.00)		16.90 (7.50, 27.10)		15.40 (7.50, 26.10)	0.117
Biochemistry parameter							
TG (mg/dL)		83.28 (66.45, 108.98)		81.51 (65.56, 105.43)		98.79 (76.20, 123.38)	< 0.001
TC (mg/dL)		159.60 ± 25.57		158.17 ± 24.67		166.52 ± 28.62	< 0.001
HDL-C (mg/dL)		58.39 (50.66, 66.90)		58.39 (51.04, 67.29)		58.20 (48.24, 65.74)	0.141
LDL-C (mg/dL)		88.55 (75.41, 103.64)		87.39 (74.25,101.32)		95.51 (82.27, 112.92)	< 0.001
AST (U/L)		17.0 (15.0, 19.0)		17.0 (15.0, 19.0)		17.0 (15.0, 20.0)	0.601
ALT (U/L)		15.0 (12.0, 20.0)		15.0 (11.0, 20.0)		15.0 (12.0, 21.0)	0.111
eGFR (mL/min per 1.73 m2 )		137.92 (124.35, 152.27)		138.56 (124.83, 152.85)		135.58 (121.90, 147.62)	0.028
UA (mg/dL)		3.66 (3.19, 4.27)		3.61 (3.18, 4.22)		3.98 (3.35, 4.56)	< 0.001
OGTT FPG (mg/dL)		77.22 ± 8.07		75.95 ± 6.63		83.34 ± 11.06	< 0.001
OGTT-1hPPG (mg/dL)		137.30 ± 29.93		129.70 ± 24.62		173.91 ± 25.99	< 0.001
OGTT-2hPPG (mg/dL)		121.95 ± 25.26		114.51 ± 18.18		157.81 ± 23.89	< 0.001

Data were expressed as the mean ± SD or median (upper and lower quartiles) or number (%). ^*^The number of participants with TPO-Ab value was 1231. ^#^The number of participants with TG-Ab value was 1098. Abbreviations: ALT, alanine aminotransferase; AST, aspartate aminotransferase; eGFR, estimated glomerular filtration rate; FPG, fasting plasma glucose; FT3, free triiodothyronine; FT4, free thyroxine; GDM, gestational diabetes mellitus; HDL-C, high-density lipoprotein cholesterol; LDL-C, low-density lipoprotein cholesterol; pBMI, pre-pregnancy body mass index; PTFQI, Parametric thyroid feedback quantile-based index; TC, total cholesterol; TFQI, Thyroid Feedback Quantile-based Index; TG, triglycerides; TG-Ab, anti-thyroglobulin antibodies; TPO-Ab, anti-thyroid peroxidase antibodies; TSH, thyroid-stimulating hormone; TSHI, TSH index; TT4RI, thyrotrophic T4 resistance index; UA, uric acid

### Characteristic of participants with GDM stratified by pBMI

We then explore the difference between GDM and non-GDM women in pre-pregnancy obese and non-obese group, separately. As shown in Table 2, in non-obese group, women with GDM exhibited older age, higher pBMI levels, higher first-trimester serum lipid levels and UA levels (all *P* < 0.001). Additionally, women with GDM had higher TFQI, PTFQI, TSHI and TT4RI levels compared to non-GDM women (all *P* < 0.05), indicating a relative insensitivity to thyroid hormone status. Whereas in the obese group, women with or without GDM had comparable pBMI levels and serum lipid levels at the first trimester. It is noteworthy that, obese women with GDM exhibited lower levels of TFQI, PTFQI, TSHI and TT4RI levels compared to those without GDM (all *P* < 0.01), indicating a relative higher sensitivity to thyroid hormone status. Correlation analysis also indicated that TFQI (r = − 0.187), PTFQI (r = − 0.182) and TSHI (r = − 0.184) were negatively associated with OGTT-FBG levels only in obese group but not non-obese group, indicating a modifying effect of pre-pregnancy obesity on their relationships (Supplementary Table 2).

**Table 2:** Characteristic of pregnant women with or without GDM in pre-pregnancy non-obese and obese subgroups

Non-obese				Obese		
**Variables**	**Non-GDM**	**GDM**	***P *** **value**	**Non-GDM**	**GDM**	***P *** **value**
*N*	960	182		125	43	
Age, years	31.27 ± 3.46	32.93 ± 4.07	< 0.001	31.97 ± 3.59	32.72 ± 4.12	0.255
Gestational week	39.33 ± 1.28	38.91 ± 1.26	< 0.001	38.99 ± 1.45	39.13 ± 0.85	0.553
pBMI (kg/m^2^)	20.69 ± 2.05	21.31 ± 1.93	< 0.001	27.37 ± 2.09	27.34 ± 2.10	0.936
Parity, *n* (%)			0.048			0.363
≥ 1	186 (19.4%)	47 (25.8%)		35 (28.0%)	9 (20.9%)	
**0**	774 (81.6%)	135 (74.2%)		90 (72.0%)	34 (79.1%)	
Thyroid parameter						
FT3 (pmol/L)	4.92 ± 0.48	4.94 ± 0.48	0.594	4.99 ± 0.51	5.01 ± 0.51	0.811
FT4 (pmol/L)	16.15 ± 1.87	16.25 ± 1.96	0.515	15.95 ± 2.04	15.49 ± 1.88	0.197
TSH (mIU/mL )	1.17 (0.72,1.74)	1.31 (0.81, 1.99)	0.028	1.57 (1.00, 2.02)	1.18 (0.87, 1.56)	0.007
FT3/FT4 ratio	0.307 ± 0.036	0.307 ± 0.036	0.903	0.315 ± 0.036	0.326 ± 0.042	0.102
**TFQI**	**-0.01** ± **0.36**	**0.05** ± **0.36**	**0.024**	**0.06** ± **0.35**	**-0.12** ± **0.26**	**0.001**
**PTFQI**	**-0.01** ± **0.35**	**0.06** ± **0.35**	**0.029**	**0.06** ± **0.33**	**-0.09** ± **0.25**	**0.001**
**TSHI**	**2.30 (1.82, 2.69)**	**2.46 (1.99, 2.85)**	**0.008**	**2.52 (2.20, 2.87)**	**2.20 (1.96, 2.38)**	**< 0.001**
**TT4RI**	**18.88 (11.53,27.27)**	**21.11 (13.86,31.35)**	**0.016**	**23.33 (16.28, 33.05)**	**17.83 (14.89,23.65)**	**0.001**
TPO-Ab (U/L)	30.00 (14.00, 41.70)	30.90 (14.00, 41.10)	0.726	30.10 (14.00, 41.00)	32.20 (14.00,41.73)	0.711
TG-Ab (U/L)	16.70 (7.50, 27.00)	15.60 (7.50, 26.00)	0.215	17.90 (7.50, 30.73)	7.50 (7.50, 30.83)	0.305
Biochemistry parameter						
TG ( mg/dL )	79.74 (63.79, 101.00)	95.69 (75.31, 121.38)	< 0.001	102.78 (80.18, 129.58)	109.86 (90.82, 131.35)	0.336
TC ( mg/dL )	157.56 ± 24.50	165.39 ± 28.82	0.001	162.96 ± 25.48	171.34 ± 27.58	0.074
HDL-C ( mg/dL )	59.17 (52.20, 67.67)	59.75 (49.21, 66.51)	0.407	51.43 (43.31, 59.94)	52.59 (44.47, 59.26)	0.926
LDL-C ( mg/dL )	86.23 (73.47, 100.16)	94.16 (78.98 108.86)	< 0.001	99.00 (82.27, 119.20)	106.92 (92.13, 125.48)	0.052
AST (U/L)	17.0 (15.0, 19.0)	17.0 (15.0, 20.0)	0.957	17.0 (15.0, 21.0)	16.5 (15.0, 19.3)	0.292
ALT (U/L)	14.0 (11.0, 19.0)	15.0 (12.0, 19.0)	0.542	16.0 (13.8, 25.5)	20.0 (15.0, 23.3)	0.36
eGFR (mL/min per 1.73	139.34 (125.94, 153.30)	137.19 (123.83, 148.53)	0.227	133.47 (119.93, 148.22)	128.76 (117.54, 137.86)	0.069
m^2^ )						
UA ( mg/dL )	3.56 (3.16, 4.10)	3.87 (3.24, 4.42)	< 0.001	4.40 (3.59, 4.96)	4.42 (3.71, 5.20)	0.403
OGTT-FPG ( mg/dL )	75.62 ± 6.39	82.01 ± 11.01	< 0.001	78.46 ± 7.79	88.95 ± 9.50	< 0.001
OGTT-1hPPG ( mg/dL )	128.79 ± 24.53	172.58 ± 25.31	< 0.001	136.73 ± 24.30	179.56 ± 28.28	< 0.001
OGTT-2hPPG ( mg/dL )	114.07 ± 18.16	157.17 ± 23.98	< 0.001	117.87 ± 18.08	160.51 ± 23.59	< 0.001

Data were expressed as the mean ± SD or median (upper and lower quartiles) or number (%). Abbreviations: ALT, alanine aminotransferase; AST, aspartate aminotransferase; eGFR, estimated glomerular filtration rate; FPG, fasting plasma glucose; FT3, free triiodothyronine; FT4, free thyroxine; GDM, gestational diabetes mellitus; HDL-C, high-density lipoprotein cholesterol; LDL-C, low-density lipoprotein cholesterol; pBMI, pre-pregnancy body mass index; PTFQI, Parametric thyroid feedback quantile-based index; TC, total cholesterol; TFQI, Thyroid Feedback Quantile-based Index; TG, triglycerides; TG-Ab, anti-thyroglobulin antibodies; TPO-Ab, anti-thyroid peroxidase antibodies; TSH, thyroid-stimulating hormone; TSHI, TSH index; TT4RI, thyrotrophic T4 resistance index; UA, uric acid

### Association of indices of sensitivity to thyroid hormone with GDM

Next, we aimed to explore the contradictory associations between GDM and the indices of sensitivity to thyroid hormones. In pre-pregnancy non-obese women, the ORs for GDM were 1.250 (95% CI 1.056–1.478), 1.240 (95% CI 1.049–1.467) and 1.197 (95% CI 1.007–1.422)) for 1 SD increase in TFQI, PTFQI and TSHI after full adjustment (Table 3, all *P* < 0.05). On the contrary, in pre-pregnancy obese women, per SD increase in these composite indices were inversely associated with GDM (TFQI: OR: 0.474, 95% CI 0.303, 0.743; PTFQI: OR: 0.494, 95% CI 0.315, 0.774; TSHI: OR: 0.517, 95% CI 0.333, 0.802, TT4RI: OR 0.468, 95% CI 0.292, 0.752) (all *P* < 0.05). Further analysis verified significant interactions between TFQI, PTFQI, TSHI, TT4RI and pre-pregnancy obesity with respect to the GDM outcome (all *P* < 0.05).

**Table 3:** Logistic regression analysis for the association between the thyroid parameters and GDM in pre-pregnancy non-obese and obese subgroups

	Model 1			Model 2		
Variable(1SD)	OR (95% CI)	*P* value	*P* for Interaction	OR (95% CI)	*P* value	*P* for Interaction
**FT3**			0.986			0.912
Non-obese	1.044 (0.890,1.225)	0.594		1.120 (0.946,1.325)	0.188	
Obese	1.041 (0.750,1.445)	0.810		1.041 (0.729,1.486)	0.825	
**FT4**			0.149			**0.048**
Non-obese	1.054 (0.900,1.235)	0.514		1.132 (0.959,1.335)	0.142	
Obese	0.795 (0.561,1.127)	0.197		**0.693 (0.470,1.021)**	**0.063**	
**TSH**			**0.001**			**0.003**
Non-obese	**1.191 (1.023,1.388)**	**0.025**		1.155 (0.980,1.362)	0.086	
Obese	**0.562 (0.372,0.850)**	**0.006**		**0.563 (0.368,0.863)**	**0.008**	
**FT3/FT4 ratio**			0.128			**0.032**
Non-obese	0.990 (0.843,1.162)	0.903		0.976 (0.823,1.158)	0.784	
Obese	1.323 (0.944,1.853)	0.104		**1.609 (1.082,2.395)**	**0.019**	
**TFQI**			**< 0.001**			**< 0.001**
Non-obese	**1.199 (1.024,1.403)**	**0.024**		**1.250 (1.056,1.478)**	**0.009**	
Obese	**0.552 (0.370,0.824)**	**0.004**		**0.474 (0.303,0.743)**	**0.001**	
**PTFQI**			**0.001**			**0.001**
Non-obese	**1.191 (1.018,1.395)**	**0.030**		**1.240 (1.049,1.467)**	**0.012**	
Obese	**0.570 (0.381,0.854)**	**0.006**		**0.494 (0.315,0.774)**	**0.002**	
**TSHI**			**< 0.001**			**0.001**
Non-obese	**1.194 (1.016,1.404)**	**0.032**		**1.197 (1.007,1.422)**	**0.041**	
Obese	**0.537 (0.357,0.807)**	**0.003**		**0.517 (0.333,0.802)**	**0.003**	
**TT4RI**			**< 0.001**			**0.001**
Non-obese	**1.186 (1.020,1.378)**	**0.026**		**1.172 (0.996,1.379)**	**0.056**	
Obese	**0.480 (0.304,0.757)**	**0.002**		**0.468 (0.292,0.752)**	**0.002**	

Model 1: crude model

Model 2: adjusted for age, parity, first-trimester TG, LDL-C, UA and eGFR.

**Fig. 2:**
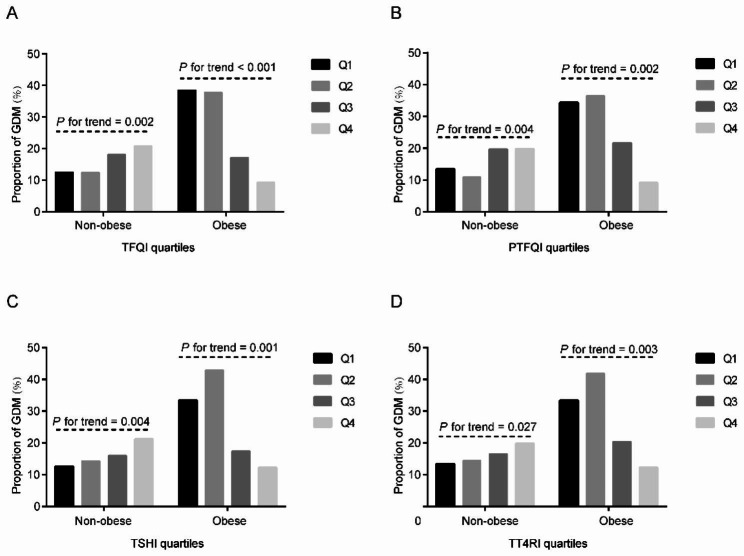
The proportion of GDM by composite indices of sensitivity to thyroid hormones. (A) the proportion of GDM across TFQI quartiles in pre-pregnancy non- obese and obese subgroups; (B) the proportion of GDM across PTFQI quartiles in pre-pregnancy non-obese and obese subgroups; (C) the proportion of GDM across TSHI quartiles pre-pregnancy non-obese and obese subgroups; (D) the proportion of GDM across TT4RI quartiles pre-pregnancy non-obese and obese subgroups. Q1, the first quartile, Q2, the second quartile, Q3, the third quartile, Q4, the fourth quartile

The composite indices of thyroid hormones were then divided into quartiles. As shown in Fig. 2, the proportion of GDM increased with increasing TFQI, PTFQI, TSHI and TT4RI quartiles in pre-pregnancy non-obese women (all *P* for trend < 0.05). However, in pre-pregnancy obese women, the prevalence of GDM exhibited decreased trend with rising quartiles of those indices (all *P* for trend < 0.05). As shown in Table 4, The Q3 to Q4 versus Q1 TFQI levels showed increasingly positive associations with GDM in non-obese women (Q3: OR 1.733, 95% CI 1.067–2.816; Q4: OR 1.981, 95% CI 1.224–3.207) (*P* for trend = 0.001). PTFQI and TSHI exhibited similar pattern. Instead, there was a negative correlation between the Q4 of TFQI, PTFQI, TSHI, TT4RI and GDM in obese women (all *P* for trend < 0.01).

**Table 4:** Logistic regression analysis for the association between the thyroid parameters quartiles and GDM in pre-pregnancy non-obese and obese subgroups

	Q1	Q2	Q3	Q4	
Variable (1SD)	OR (95% CI)	OR (95% CI)	OR (95% CI)	OR (95% CI)	*P* for trend
**TFQI**					
Non-obese	Ref.	0.955 (0.567, 1.608)	**1.733 (1.067, 2.816)** ^*^	**1.981 (1.224, 3.207)** ^**^	**0.001**
Obese	Ref.	0.926 (0.332, 2.581)	**0.298 (0.098, 0.907)** ^*^	**0.131 (0.036, 0.472)** ^**^	**< 0.001**
**PTFQI**					
Non-obese	Ref.	0.774 (0.455,1.318)	**1.776 (1.111, 2.840)** ^*^	**1.738 (1.078, 2.803)** ^*^	**0.002**
Obese	Ref.	1.091 (0.386, 3.086)	0.463 (0.149, 1.441)	**0.171 (0.046, 0.631)** ^**^	**0.002**
**TSHI**					
Non-obese	Ref.	1.099 (0.667, 1.812)	1.335( 0.817, 2.182)	**1.860 (1.156, 2.994)** ^*^	**0.008**
Obese	Ref.	1.701 (0.553, 5.231)	0.488 (0.139, 1.709)	**0.252 (0.069, 0.927)** ^*^	**0.007**
**TT4RI**					
Non-obese	Ref.	1.036 (0.636, 1.689)	1.215 (0.751, 1.966)	1.554 (0.967, 2.497)^#^	**0.045**
Obese	Ref.	1.534 (0.506,4.655)	0.668 (0.212, 2.103)	**0.253 (0.071, 0.897)** ^*^	**0.003**

^*^P < 0.05, ^**^P < 0.01, ^#^*P* = 0.068

Model was adjusted for age, parity, first-trimester TG, LDL-C, UA and eGFR.

## Discussion

Our study yielded the following results. The indices of thyroid hormone sensitivity, TFQI, PTFQI and TSHI, during early pregnancy, were positively associated with the occurrence of GDM in pre-pregnancy non-obese women, while were negatively associated with GDM in pre-pregnancy obese women. Our results suggested a modifying effect of pre-pregnancy obesity on the association between early pregnancy thyroid hormone homeostasis and GDM.

Although thyroid hormone is precisely regulated through the hypothalamic–pituitary–thyroid (HPT) axis, circulating concentration cannot fully reflect its actual effect [38, 39]. For example, some patients with hypothyroidism still show clinical symptoms, despite reaching the biochemical therapy targets after LT4 treatment, which was associated with the transition of FT4 to FT3 and the sensitivity of thyroid hormone [39]. In this study, four composite indices were adopted to evaluate central sensitivity to thyroid hormones. The TFQI and PTFQI were new indices proposed in recent years and were more accurate and stable in evaluating sensitivity to thyroid hormones compared to TSHI and TT4RI, which would be biased by the extreme value of FT4 and TSH [17]. They were closely related to adverse metabolic disorders, especially diabetes, as revealed in recent studies [17, 19]. However, only limited studies have explored their association with pregnancy outcomes.

Plenty of studies have confirmed the regulating role of the thyroid system on glucose homeostasis and GDM, although with inconsistent conclusions [4, 11]. Here, we uncovered contrary relationships between the indices of sensitivity to thyroid hormones and GDM under different pBMI status. This novel discovery indicated the complex interactions between pre-pregnancy energy status, hormone homeostasis and metabolic phenotype. Paradoxical results were also observed in non-pregnant population. Although studies have confirmed that reduced sensitivity to thyroid hormones (increased composite indices) was positively associated with diabetes and diabetes-related death [17, 19], no association between TFQI and new-onset diabetes was observed [19]. Moreover, one study indicated a protective role of reduced sensitivity to thyroid hormones on pre-diabetes in non-pregnant population [40]. In line with our findings under non-obese status, both FT4 and TSH have been shown to be associated with a higher risk of GDM [7, 25, 26, 41, 42]. However, an adverse relationship was observed under obese status. To our knowledge, there was only one study addressed a negative link between TFQI and GDM. However, they did not explore the modifying effect of pBMI [24]. In GDM women of non-obese group, reduced sensitivity to thyroid hormone was concomitant with a worse metabolic phenotype such as hyperlipidemia, higher BMI levels during early gestation. However, it appears that reduced sensitivity to thyroid hormones was an adaptable protective factor against energy oversupply under obese status, as the early pregnancy metabolic parameters among GDM and non-GDM subjects were comparable in addition to reduced resistance to thyroid hormones. Furthermore, most previous studies indicated a protective role of FT4 in the occurrence of GDM, indicating a complex modulation effect of thyroid homeostasis on glucose metabolism [4, 11, 26]. The discrepancy in both previous studies and our results may be attributed to the dual effects of thyroid hormones on glucose homeostasis, as it could not only lower glucose levels by increasing glucose utilization but also up regulate glucose levels by stimulating hepatic glycogenolysis and glucose intestinal absorption [24]. It is worth noting that, compared to non-pregnant population with diabetes, women with GDM had relatively less impaired glycemic control and energy imbalance. Collectively, further investigation is warranted for analyzing the contrary relationships in pregnant population.

Thyroid homeostasis itself could be modulated by obesity [43, 44]. For instance, the elevated TSH levels in obese premenopausal women could be reversed by weight loss or bariatric surgery [43]. Previous studies revealed inconsistent associations between thyroid hormone sensitivity and obesity. Some studies indicated a positive link of TFQI to obesity, while others indicated a reverse association between TFQI and BMI [17, 19, 45]. However, neither TFQI nor PTFQI but only TSHI and TT4RI were increased in obese women in our study. Iodine status may be a linker between pre-pregnancy energy status and thyroid hormone sensitivity, as previous studies have indicated a causal role of obesity for iodine deficiency. Previous studies also demonstrated compensated elevated thyroid sensitivity under iodine deficiency status [46, 47]. More importantly, iodine deficiency was associated with the occurrence of GDM [48]. Existing studies have also documented that adipocyte hormone such as leptin may be an underline modulator on the HPT axis under obesity status [24, 43].Therefore, further studies are warranted to take into account iodine status and hormones.

Plenty of evidence has documented causal effects of maternal thyroid homeostasis, pBMI and GDM on both obstetric complications and fetal health outcomes [49–53]. Hence, it is of vital significance to explore the pregnancy outcomes of GDM women with different thyroid hormone sensitivity and pBMI levels, which may deepen our understanding of GDM pathogenesis and add further guides for individualized GDM management. For example, previous study has indicated that GDM patients with underweight BMI encountered lower risk of preeclampsia and macrosomia and required a relaxed maternal glycemic target [54].

There are several limitations in our study. Firstly, this is a single-center retrospective study limited by small sample size and selection bias. Secondly, some confounders, such as socio-demographic characteristics, education, income levels and iodine intake, were not collected [46, 55]. Thirdly, there may be bias in pBMI as it was calculated based on self-reported pre-pregnancy body weight from pregnant women. Lastly, more than one measurement of maternal thyroid function could more accurate considering the variance in maternal thyroid hormones during the first trimester. Taken together, it will be necessary to validate our findings in more large-scale prospective studies.

## Conclusions

In conclusion, this study found contrary associations between sensitivity to thyroid hormones and GDM modified by pre-pregnancy obesity. Our novel findings suggested complicated interactions between pBMI and thyroid hormone sensitivity on maternal complications and possibly fetal growth. More future studies are warranted to verify our findings and to further explore its influence on both maternal and fetal consequences.

### Electronic supplementary material

Below is the link to the electronic supplementary material.

Supplementary Material 1

## Data Availability

Some or all datasets generated during and/or analyzed during the current study are not publicly available but are available from the corresponding author on reasonable request.

## Abbreviations

ALT: Alanine aminotransferase
AST: aspartate aminotransferase
BMI: body mass index
CTD: connective tissue disease
eGFR: estimated glomerular filtration rate
FT3: free triiodothyronine
FT4: free thyroxine
GDM: gestational diabetes mellitus
HDL-C: high-density lipoprotein cholesterol
LDL-C: low-density lipoprotein cholesterol
NAFLD: nonalcoholic fatty liver disease
OR: odds ratio
pBMI: pre-pregnancy BMI
PCOS: polycystic ovary syndrome
PTFQI: parametric TFQI
TC: total cholesterol
TFQI: thyroid feedback quantile-based index
TG: triglyceride
TSH: thyroid-stimulating hormone
TSHI: thyroid-stimulating hormone index
TT4RI: thyrotrophic T4 resistance index
TG-Ab: anti-thyroglobulin antibodies
TPO-Ab: anti-thyroid peroxidase antibody
