# Supplementary material for: Maternal pre-pregnancy obesity modifies the association between first-trimester thyroid hormone sensitivity and gestational Diabetes Mellitus: a retrospective study from Northern China
Source: Diabetol Metab Syndr. 2023 Oct 25;15:212. doi: 10.1186/s13098-023-01188-6 (PMC10598956; doi:10.1186/s13098-023-01188-6)
Supplement: Supplementary file 1 — Supplementary Material 1 [file 13098_2023_1188_MOESM1_ESM.docx]

**Maternal pre-pregnancy obesity modifies the association between first-trimester thyroid hormone sensitivity and gestational diabetes mellitus: A retrospective study from Northern China**

**Honglin Sun^1^†, Yibo Zhou^1^†, Jia Liu^1^, Ying Wang^2^*, Guang Wang^1^***

^1^Department of Endocrinology, Beijing Chao-yang Hospital, Capital Medical University, Beijing 100020, China;

^2^Physical Examination Center, Beijing Chao-Yang Hospital, Capital Medical University, Beijing, China.

*Correspondence:

Ying Wang

Email address: hdwangying0517@163.com

Guang Wang

Email address:wangguang@bjcyh.com

†These authors have contributed equally to this work

## Supplementary Materials

## Supplementary Table 1. Basic characteristic of the whole population in pre-

**pregnancy obese and non-obese subgroups.**

| **Variables** |  | **Total** | |  | **Non-obese** | | |  | **Obese** | | ***P* value** | |  |
| --- | --- | --- | --- | --- | --- | --- | --- | --- | --- | --- | --- | --- | --- |
| *N* | |  | 1310 | | |  | 1142 | |  | 168 | | - | |
| Age (years) | |  | 31.62 ± 3.63 | | |  | 31.54 ± 3.61 | |  | 32.16 ± 3.73 | | 0.038 | |
| Gestational time (week) | |  | 39.23 ± 1.29 | | |  | 39.26 ± 1.28 | |  | 39.03 ± 1.32 | | 0.027 | |
| pBMI (kg/m^2^) | |  | 21.63 ± 3.00 | | |  | 20.79 ± 2.04 | |  | 27.36 ± 2.09 | | < 0.001 | |
| Parity, *n* (%) | |  |  | | |  |  | |  |  | | 0.086 | |
| ≥1 | |  | 277 (21.1%) | | |  | 233 (20.4%) | |  | 44 (26.2%) | |  | |
| 0 | |  | 1033 (78.9%) | | |  | 909 (79.6%) | |  | 124 (73.8%) | |  | |
| Thyroid parameter | |  |  | | |  |  | |  |  | |  | |
| FT3 (pmol/L) | |  | 4.93 ± 0.49 | | |  | 4.92 ± 0.48 | |  | 4.99 ± 0.51 | | 0.095 | |
| FT4 (pmol/L) | |  | 16.12 ± 1.90 | | |  | 16.16 ± 1.88 | |  | 15.83 ± 2.00 | | 0.034 | |
| TSH (μIU/mL ) | |  | 1.22 (0.75,1.79) | | |  | 1.19 (0.73, 1.78) | |  | 1.47 (0.97, 1.90) | | 0.002 | |
| FT3/FT4 ratio | |  | 0.309 ± 0.037 | | |  | 0.307 ± 0.036 | |  | 0.318 ± 0.038 | | < 0.001 | |
| **TFQI** | |  | **0.00 ± 0.36** | | |  | **0.00 ± 0.36** | |  | **0.02 ± 0.34** | | **0.521** | |
| **PTFQI** | |  | **0.01 ± 0.35** | | |  | **0.00 ± 0.35** | |  | **0.02 ± 0.32** | | **0.457** | |
| **TSHI** | |  | **2.35 (1.89, 2.72)** | | |  | **2.34 (1.86, 2.71)** | |  | **2.39 (2.09, 2.78)** | | **0.020** | |
| **TT4RI** | |  | **19.58 (12.00, 27.97)** | | |  | **19.21 (11.64, 27.77)** | |  | **22.27 (15.51, 29.89)** | | **0.006** | |
| TPO-Ab (U/L)^*^ | |  | 30.10 (14.00, 41.70) | | |  | 30.10 (14.00, 41.70) | |  | 30.40 (14.00, 40.98) | | 0.876 | |
| TG-Ab (U/L)^#^ | |  | 16.55 (7.50, 27.00) | | |  | 16.55 (7.50, 26.63) | |  | 16.65 (7.50, 30.73) | | 0.844 | |
| Biochemistry parameter | |  |  | | |  |  | |  |  | |  | |
| TG (mg/dL) | |  | 83.28 (66.45, 108.98) | | |  | 81.51 (64.68, 105.43) | |  | 105.43 (82.40, 130.02) | | < 0.001 | |
| TC (mg/dL) | |  | 150.60 ± 25.57 | | |  | 158.80 ± 25.39 | |  | 165.11±26.21 | | 0.003 | |
| HDL-C (mg/dL) | |  | 58.39 (50.66, 66.90) | | |  | 59.17 (52.20, 67.29) | |  | 51.62 (43.50, 59.36) | | < 0.001 | |
| LDL-C (mg/dL) | |  | 88.55 (75.41, 103.64) | | |  | 87.01 (74.25, 101.32) | |  | 100.35 (85.07, 120.94) | | < 0.001 | |
| AST (U/L) | |  | 17.0 (15.0, 19.0) | | |  | 17.0 (15.0, 19.0) | |  | 17.0 (15.0, 20.0) | | 0.894 | |
| ALT (U/L) | |  | 15.0 (12.0, 20.0) | | |  | 14.0 (11.0, 19.0) | |  | 17.0 (14.0, 25.0) | | < 0.001 | |
| eGFR (mL/min per 1.73  m^2^ ) | |  | 137.92 (124.35, 152.27) | | |  | 138.95 (125.56, 152.82) | |  | 131.03 (119.14, 145.75) | | < 0.001 | |
| UA (mg/dL) | |  | 3.66 (3.19, 4.27) | | |  | 3.60 (3.18, 4.17) | |  | 4.40 (3.64, 4.96) | | < 0.001 | |
| OGTT FPG (mg/dL) | |  | 77.22 ± 8.07 | | |  | 76.64 ± 7.68 | |  | 81.14 ± 9.43 | | < 0.001 | |
| OGTT-1hPPG (mg/dL) | |  | 137.30 ± 29.93 | | |  | 135.77 ± 29.40 | |  | 147.69 ± 31.48 | | < 0.001 | |
| OGTT-2hPPG (mg/dL) | |  | 121.95 ± 25.26 | | |  | 120.94 ± 24.85 | |  | 128.79 ± 27.04 | | < 0.001 | |
| GDM, *n* (%) | |  |  | | |  |  | |  |  | | 0.002 | |
| Yes | |  | 225 (17.2%) | | |  | 182 (15.9%) | |  | 43 (25.6%) | |  | |
| No | |  | 1085 (82.8%) | | |  | 960 (84.1%) | |  | 125 (74.4%) | |  | |

Data were expressed as the mean ± SD or median (upper and lower quartiles) or number (%). ^*^The number of participants with TPO-Ab value was 1231. ^#^The number of participants with TG-Ab value was 1098. Abbreviations: ALT, alanine aminotransferase; AST, aspartate aminotransferase; eGFR, estimated glomerular filtration rate; FPG, fasting plasma glucose; FT3, free triiodothyronine; FT4, free thyroxine; GDM, gestational diabetes mellitus; HDL-C, high-density lipoprotein cholesterol; LDL-C, low-density lipoprotein cholesterol; pBMI, pre-pregnancy body mass index; PTFQI, Parametric thyroid feedback quantile-based index; TC, total cholesterol; TFQI, Thyroid Feedback Quantile-based Index; TG, triglycerides; TG-Ab, anti-thyroglobulin antibodies; TPO-Ab, anti-thyroid peroxidase antibodies; TSH, thyroid-stimulating hormone; TSHI, TSH index; TT4RI, thyrotrophic T4 resistance index; UA, uric acid.

## Supplementary Table 2. Correlation between thyroid parameters and OGTT

**plasma glucose levels pre-pregnancy obese and non-obese subgroups.**

| **Outcome** |  | **Total** |  | **Non-obese** |  | **Obese** |
| --- | --- | --- | --- | --- | --- | --- |
| **OGTT-FPG** |  |  |  |  |  |  |
| FT3 |  | 0.054 |  | 0.050 |  | 0.030 |
| FT4 |  | -0.034 |  | 0.001 |  | -0.148 |
| TSH |  | 0.031 |  | 0.035 |  | -0.108 |
| FT3/FT4 ratio |  | **0.083*** |  | 0.043 |  | **0.187*** |
| TFQI |  | 0.013 |  | 0.043 |  | **-0.187*** |
| pTFQI |  | 0.014 |  | 0.043 |  | **-0.182*** |
| TSHI |  | 0.015 |  | 0.029 |  | **-0.184*** |
| TT4RI |  | 0.026 |  | 0.037 |  | -0.147 |
| **OGTT-1hPPG** |  |  |  |  |  |  |
| FT3 |  | 0.049 |  | 0.021 |  | **0.172*** |
| FT4 |  | -0.004 |  | 0.002 |  | 0.012 |
| TSH |  | 0.021 |  | 0.030 |  | -0.133 |
| FT3/FT4 ratio |  | 0.041 |  | 0.011 |  | 0.139 |
| TFQI |  | 0.019 |  | 0.027 |  | -0.058 |
| pTFQI |  | 0.021 |  | 0.028 |  | -0.048 |
| TSHI |  | 0.023 |  | 0.032 |  | -0.130 |
| TT4RI |  | 0.021 |  | 0.031 |  | -0.138 |
| **OGTT-2hPPG** |  |  |  |  |  |  |
| FT3 |  | 0.033 |  | 0.022 |  | 0.067 |
| FT4 |  | 0.005 |  | 0.029 |  | -0.091 |
| TSH |  | 0.024 |  | 0.027 |  | -0.095 |
| FT3/FT4 ratio |  | 0.022 |  | -0.011 |  | 0.150 |
| TFQI |  | 0.025 |  | 0.049 |  | -0.151 |
| pTFQI |  | 0.029 |  | 0.050 |  | -0.133 |
| TSHI |  | 0.034 |  | 0.044 |  | -0.102 |
| TT4RI |  | 0.026 |  | 0.037 |  | -0.120 |

**P* < 0.05. TSH, TSHI and TT4RI were log transformed for analysis due to skewed distribution.
